# Exploring the therapeutic potential of precision medicine in rare genetic obesity disorders: a scientific perspective

**DOI:** 10.3389/fnut.2024.1509994

**Published:** 2024-12-23

**Authors:** Tinh-Hai Collet, Valerie Schwitzgebel

**Affiliations:** ^1^Service of Endocrinology, Diabetes, Nutrition, and Therapeutic Education, Geneva University Hospitals, Geneva, Switzerland; ^2^Faculty of Medicine, Diabetes Center, University of Geneva, Geneva, Switzerland; ^3^Pediatric Endocrine and Diabetes Unit, Department of Pediatrics, Obstetrics, and Gynecology, Geneva University Hospitals, Geneva, Switzerland; ^4^Institute of Genetics and Genomics in Geneva (iGE3), University of Geneva, Geneva, Switzerland

**Keywords:** monogenic obesity, melanocortin-4 receptor (MC4R), proprotein convertase subtilisin/kexin-type 1 (PCSK1), pro-opio-melanocortin (POMC), leptin receptor (LEPR), leptin-melanocortin pathway, Bardet-Biedel syndrome, precision medicine

## Abstract

The prevalence of obesity is increasing worldwide, affecting both children and adults. This obesity epidemic is mostly driven by an increase in energy intake (abundance of highly palatable energy-dense food and drinks) and to a lesser degree a decrease in energy expenditure (sedentary lifestyle). A small proportion of individuals with obesity are affected by genetic forms of obesity, which often relate to mutations in the leptin-melanocortin pathway or are part of syndromes such as the Bardet-Biedl syndrome. These rare forms of obesity have provided valuable insights into the genetic architecture of obesity. Recent advances in understanding the molecular mechanisms that control appetite, hunger, and satiety have led to the development of drugs that can override genetic defects, enabling precision treatment. Leptin deficiency is uniquely treated with recombinant human metreleptin, while those with LEPR, PCSK1, or POMC deficiency can now be treated with the MC4R agonist setmelanotide. This review highlights the most frequent monogenic and syndromic forms of obesity, and the future outlook of precision treatment for these conditions.

## Introduction

As global obesity prevalence continues to climb, rare forms of obesity remain underdiagnosed and insufficiently recognized, despite their classification as orphan diseases. This review delves into the epidemiology of common and rare obesity, highlighting the underlying mechanisms. It also explores recent advances in targeted therapies, such as the melanocortin 4 receptor (MC4R) agonist setmelanotide, underscoring the critical need for personalized approaches to address these unique and often overlooked conditions effectively.

### Epidemiology

One in eight people is affected by obesity worldwide, translating into over 1 billion people globally, including approximately 890 million adults, 160 million children and adolescents (aged 5–19 years) (1). When adding those who are overweight, the numbers are far greater with an estimated 43% of adults and 18% children with overweight in 2022.

Hundreds of genes have been associated with obesity-related traits, while fewer genes are recognized as causally implicated in obesity (2–5). In terms of causal genes, around 20–30 genes have been identified as having a clear role in the development of monogenic forms of obesity. A small proportion of individuals are affected by genetic forms of obesity (3.9–9.3%) (6), as first highlighted by twin studies among adults (7, 8) and in childhood (9) showing a high heritability of body mass index (BMI). Monogenic forms of obesity are rare disorders, some of which are registered as orphan diseases. While being rare taken individually, collectively they can affect up to 5–7% of children with severe obesity (10, 11). In this review, we explore forms of monogenic obesity for which precision treatments are now available.

## Monogenic and syndromic forms of obesity

### Monogenic obesity

The most common monogenic form of obesity is associated with mutations in the *MC4R* gene (12), followed by mutations in the *LEPR*, *POMC*, *PCSK1*, and *LEP* genes. The prevalence of loss of function *MC4R* variants in the UK population is estimated at 1 in 340 (13). This prevalence rises to 0.5–1.7% among obese adults (BMI > 30 kg/m^2^) and around 5% in those with severe obesity (12–15). In cases of severe childhood-onset obesity, the prevalence can be even higher, varying by ethnic group (16, 17). The specific variant is also significant; highly pathogenic variants typically result in early childhood obesity, while variants with milder effects may contribute to common polygenic obesity. In addition to monogenic obesity, certain syndromes are linked to obesity. Bardet-Biedl Syndrome (BBS) has an estimated prevalence of 1 in 160,000 in northern Europe, 1 in 100,000 in the U.S., and 1 in 13,500 in some Middle Eastern populations (18). Although epidemiological data is limited in Europe, Denmark has an estimated prevalence of 1 in 59,000, while Reunion Island, France, reports rates of 1 in 45,000 to 66,000, likely due to a founder effect. Alström Syndrome (ALMS), caused by homozygous or compound heterozygous mutations in the *ALMS1* gene, has a prevalence of approximately 1 in 1,000,000.^1^ However, higher frequencies have been reported in populations with high consanguinity or geographic isolation, with over 950 cases identified worldwide.

### Genes involved in the leptin-melanocortin pathway

**Figure 1:**
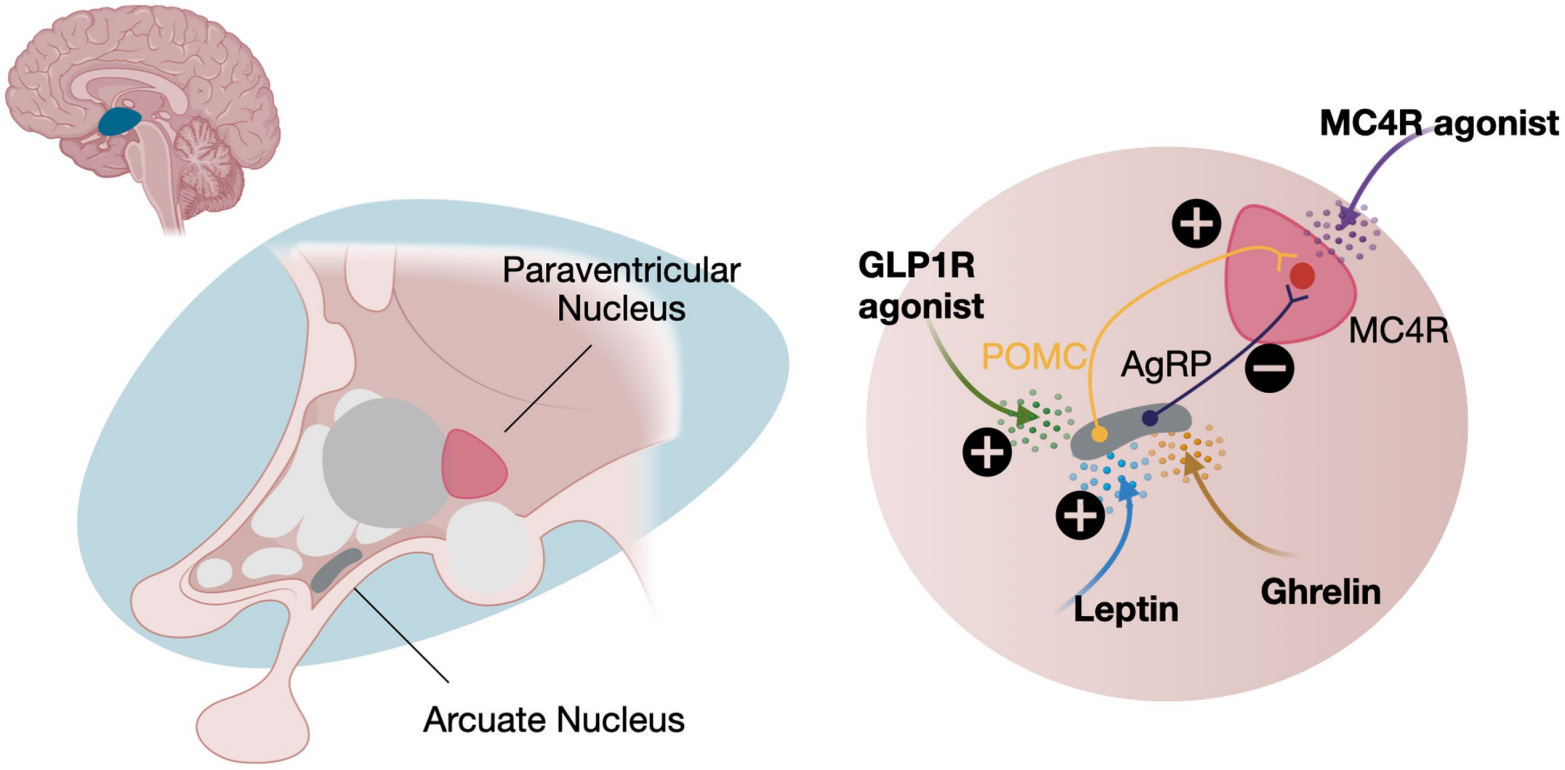
The central role of the leptin-melanocortin pathway. Leptin and ghrelin have opposing effects on appetite regulation. Leptin inhibits appetite by activating POMC neurons, which stimulate MC4R. In contrast, ghrelin, secreted by the stomach during fasting, activates AgRP neurons, inhibiting MC4R signaling and increasing appetite. Treatment with either a GLP1R or a MC4R agonists can help decrease appetite. POMC, Pro-opio-melanocortin expressing neurons; AgRP, Agouti related-protein expressing neurons; MC4R, Melanocortin receptor expressing neurons; GLP1R, Glucagon-like peptide 1 receptors. Created in BioRender. Schwitzgebel, V. (2024) https://BioRender.com/y38z865

Leptin, produced by adipocytes, correlates with body fat and serves as a key signal for the hypothalamic arcuate nucleus. Here, it stimulates pro-opio-melanocortin (POMC) expression, which is cleaved into α- and β-melanocyte-stimulating hormones (MSH) (19). These hormones act on neurons in the paraventricular nuclei to reduce appetite and increase fat oxidation via the sympathetic nervous system. The leptin-melanocortin pathway is central to energy metabolism and body weight regulation. Mutations in *MC4R* or upstream genes, discussed below, can disrupt α- and β-MSH functions, leading to increased energy intake and early-onset obesity (12) (Figure 1).

#### Leptin and leptin receptor

The human leptin gene (*LEP*) is located on chromosome 7q32.1 and encodes the 16 kDa hormone leptin, which has a four-helix bundle structure typical of cytokines. Leptin binding to its receptor triggers dimerization and activates the JAK–STAT signaling pathway, influencing metabolism, appetite, and energy expenditure (20). Mutations in *LEP* can lead to congenital leptin deficiency, resulting in severe obesity (21). The leptin receptor is encoded by the *LEPR* gene on chromosome 1p31.3 and exists in multiple isoforms, with the long form (Ob-Rb) being crucial for leptin signaling in the hypothalamus (22). Mutations in *LEPR* can cause receptor deficiency, leading to severe obesity and hyperphagia due to improper leptin signaling (23, 24).

#### Pro-opiomelanocortin

The *POMC* gene, located on chromosome 2p23.3, encodes a precursor, pro-opiomelanocortin, that is processed into several peptides involved in energy homeostasis, adrenal function, and pigmentation. POMC is mainly expressed in the anterior pituitary, hypothalamus, and skin, and is cleaved into active peptides such as adrenocorticotropic hormone (ACTH), α−/β-MSH, and β-endorphin by specific prohormone convertases (such as PCSK1 and PCSK2). α-MSH is crucial for appetite suppression, while β-endorphin modulates pain and reward pathways. Mutations in *POMC* can lead to early-onset obesity, adrenal insufficiency, and pigmentation disorders (25).

#### Proprotein convertase subtilisin/kexin-type 1

The *PCSK1* gene on chromosome 5q15-q21 encodes an enzyme vital for converting prohormones into active forms. Primarily expressed in neuroendocrine cells of the pancreas, intestines, and brain, PCSK1 processes key hormones like insulin, glucagon, and POMC, which are crucial for glucose metabolism, energy balance, and appetite regulation. Mutations can result in enzyme deficiency, leading to obesity, hyperphagia, and endocrine dysfunction (26, 27).

#### Melanocortin 4 receptor

The *MC4R* gene, located on chromosome 18q21.32, encodes a G protein-coupled receptor essential for energy homeostasis and appetite regulation, primarily in the hypothalamus (28). MC4R mediates the effects of neuropeptides from POMC, and when α-MSH binds, it activates G proteins that reduce food intake and increase energy expenditure (29, 30). *MC4R* variants also affect endocytosis, trafficking and dimerization highlighting various cellular mechanisms in weight regulation. Conversely, the agouti-related peptide acts as an antagonist, blocking α-MSH binding and promoting increased appetite and reduced energy expenditure. Mutations in *MC4R* or upstream signaling, can prevent α- and β-MSH from exerting their effects, leading to increased energy intake and weight gain from early childhood and into adulthood (12).

### Syndromic forms of obesity

#### Bardet-Biedl syndrome (BBS)

BBS is a heterogeneous disorder caused by mutations in over 25 different genes (31). While the mechanism underlying hyperphagia in BBS remains unclear, reduced ciliary length impairs leptin signaling (32). Cilia are essential sensory organelles on the surface of POMC neurons, and studies show that ciliary defects in specific hypothalamic neurons can induce obesity and hyperphagia in mice (33). BBS is characterized by six primary features: retinal degeneration, truncal obesity, postaxial polydactyly, hypogonadism, intellectual disability, and renal abnormalities (34). Obesity is a prominent feature, affecting 72–92% of patients, with significant weight gain typically observed early in life. By age 2, 33% of children are overweight and 23% are obese; by age 5, 90% are either overweight or obese (31). Additionally, the prevalence of type 2 diabetes among adolescents with BBS is 6% (35), along with hypertension and hypertriglyceridemia, which increase the risk of cardiovascular disease (36, 37).

#### Alström syndrome (ALMS)

ALMS is caused by mutations in the *ALMS1* gene, located on chromosome 2p13.1. This gene is essential for cilia function—hair-like structures on cell surfaces that play critical roles in signaling and sensory functions. Mutations in *ALMS1* lead to a rare genetic disorder characterized by progressive vision and hearing loss, obesity, type 2 diabetes, heart disease, and kidney dysfunction, among other symptoms (38).

## Hyperphagia, hunger and satiety

Hyperphagia is a common feature of all monogenic and syndromic forms of obesity (39). It is characterized by an abnormally intense and persistent sensation of hunger or urge to eat, often leading to overeating. Unlike typical hunger, this condition does not diminish after eating, frequently resulting in the rapid consumption of excessive amounts of food. Hyperphagia is not a disorder in itself but a symptom of an underlying medical issue, such as a genetic disruption in the leptin-melanocortin signaling pathway (40). This pathway plays a key role in the homeostatic regulation of eating, as opposed to the hedonic pathway, which is associated with the more common polygenic form of obesity.

Often confused with hyperphagia but distinct from it, hunger is a sensation that drives the consumption of food. Hunger typically arises a few hours after eating and is generally considered unpleasant.

Satiety, which usually occurs 15–20 min after eating, is a state of fullness that extends beyond mere satisfaction, representing the opposite of hunger. After satiation (the point at which a meal ends), satiety persists as a feeling of fullness until the next meal (41). When food is still present in the gastrointestinal tract after a meal, satiety signals suppress hunger signals, but as time passes, satiety gradually fades while hunger increases.

### How to measure hunger: different hunger scales

Like other patient-reported outcomes such as pain and fatigue, hunger can only be assessed through self-report rather than clinical or laboratory evaluations. A widely used tool for this purpose is the visual analog scale (VAS), which features a horizontal line, usually 100 mm long, with endpoints labeled “Not at all hungry” and “Extremely hungry” (42). Patients mark or slide along this line to indicate their hunger level, providing a quantifiable score that is easy to administer, reproducible, and sensitive to short-term changes, much like VAS tools for other symptoms. Another popular tool is the Likert scale, where participants rate statements on hunger or satiety using a graded scale (e.g., 1–5 or 1–7) (41, 43).

### Burden on families and patients

Numerous studies highlight the substantial burden of genetically related hyperphagia on patients and caregivers, particularly in cases involving POMC, PCSK1, and LEPR deficiencies, as well as ALMS and BBS (44). Patients often experience intense emotional distress, including sadness, frustration, anxiety, and guilt, driven by a relentless preoccupation with food and an inability to control their hunger (45). Caregivers, especially parents, share this emotional strain, facing feelings of guilt, helplessness, and frustration as they navigate their child’s behaviors, such as food sneaking and hoarding (46). The persistent focus on food intrudes on daily life, affecting patients’ school and work performance while limiting social engagement. This ongoing challenge severely diminishes their quality of life, as highlighted in a multi-country survey (47). These findings underscore the multifaceted burden that hyperphagia places on patients, siblings and caregivers, highlighting the urgent need for precision therapies to address this debilitating condition.

## Need of early detection and management: screening program, genetic confirmation, to decrease complications

In patients with early-onset obesity, especially when it begins before the age of 5, physicians should strongly consider the possibility of a genetic cause (48). Pediatricians play a key role in identifying cases of monogenic obesity, using characteristic BMI trajectories as a diagnostic aid, since these patterns differ from those seen in polygenic obesity (49). For confirmation and expert evaluation, referral to tertiary obesity clinics is recommended, where specialists have the expertise to conduct and interpret the necessary genetic tests, such as dedicated obesity gene panels or whole exome/genome sequencing. The French INSERM NutriOmics group has developed an online diagnostic support tool called ObsGen to help practitioners diagnose monogenic obesity more effectively (50).^2^ Early treatment of patients with genetic obesity is crucial, as it helps to limit the condition’s progression during adolescence, prevents related complications (51), and reduces the stigmatization and suffering these individuals often experience (52).

### Lack of response to conventional therapies—failure of diet and lifestyle interventions, no sustained response to bariatric surgery

Patients with mutations in the leptin-melanocortin pathway and BBS often receive dietary and exercise counseling similar to those with polygenic obesity, yet they show limited response to these lifestyle changes, as seen in MC4R deficiency (53). While bariatric surgery is effective for common obesity (54), its success relies on a functional leptin-melanocortin pathway (55, 56). Thus, alternative treatments are necessary for this genetic population, as bariatric surgery is typically ineffective unless carefully considered for select adults (57, 58).

Glucagon-like peptide-1 (GLP-1) receptor agonists (RAs) show promise in monogenic and syndromic obesity, with real-world evidence of effectiveness in ALMS and BBS patients (59, 60). A study of liraglutide (3.0 mg for 16 weeks) reported weight loss in patients with MC4R deficiency (6.8 kg ± 1.8 kg) compared to controls (6.1 kg ± 1.2 kg) (61), suggesting that GLP-1 RAs may work independently of a fully functional leptin-melanocortin pathway. Ongoing trials of newer GLP-1 RAs and dual/triple agonists are awaited for further insights.

## Precision treatment approach

### Metreleptin

Leptin deficiency due to *LEP* mutations is uniquely treated with recombinant human leptin (metreleptin). This synthetic analog is administered subcutaneously at 0.03 mg/kg of lean body mass daily, leading to significant weight loss and reduced hyperphagia (62, 63). In one case, a 9-year-old patient lost 16.4 kg in the first year and achieved BMI reduction over 4 years, despite weight remaining above the 98th percentile by age 14 (62, 63). The development of metreleptin-neutralizing antibodies can lead to hyperphagia recurrence and weight regain. This therapy is ineffective for patients with downstream leptin-melanocortin pathway mutations.

### Setmelanotide

Setmelanotide, a synthetic cyclic peptide, binds with high affinity to human MC4R (64). Given the central role of the leptin-melanocortin pathway, this MC4R agonist can effectively “rescue” downstream signaling, even without upstream LEPR, PCSK1, or POMC activity. However, treatment is appropriate only for cases with validated loss-of-function mutations — either homozygous or specific heterozygous autosomal dominant mutations of *LEPR*, *PCSK1* or *POMC*. Missense mutations with neutral or partial deleterious effects are unlikely to benefit (29, 30, 65, 66). This approach heralds a future of precision medicine, where treatment is tailored to variant-specific responses based on genotype.

### What are the therapeutic goals of setmelanotide?

The therapeutic goals of setmelanotide for treating obesity linked to POMC, PCSK1, or LEPR deficiencies and BBS include weight stabilization, hyperphagia control, quality of life improvement, safety and tolerability, enhanced metabolic and cardiovascular health, and sustained long-term efficacy.

### Efficacy of setmelanotide

In infants and children, weight stabilization—not weight loss—is prioritized to prevent adverse effects on growth (24, 67–70). Weight control is achieved primarily by reducing hyperphagia, as children with *LEPR* and *POMC* mutations consume three times more calories per kg of lean mass than controls (24). Additionally, children with homozygous *MC4R* mutations consume more than those with partial loss-of-function variants (64). Setmelanotide has shown promising results in clinical trials and in countries where it is marketed. So far, a relatively small number of patients have been tested with this MC4R agonist, but setmelanotide efficiently leads to weight control in patients with LEPR and POMC deficiencies and BBS (71, 72) (Table 1). Hunger reduction has been measured on an 11-point Likert scale, though some studies used retrospective self-reports, introducing potential recall bias (45, 46). Nonetheless, most studies indicate a reduction in hyperphagia with setmelanotide (46, 73).

**Table 1:** Summary of studies on setmelanotide efficacy.

Author, year	Population	Intervention	Outcomes, mean (SD)
LEPR and POMC deficiency			
(71) ClinicalTrials.gov NCT03287960	LEPR (*n* = 11) deficiency, Hom or compound Het 50% female Age at inclusion ≥6 years Mean age 23.7 years (SD 8.4) Of whom, 3 were younger than 18 years Obesity (>95th percentile, or adult BMI ≥ 30 kg/m^2^) Mean BMI 48.2 kg/m^2^ (SD 10.4) in adults Mean BMI Z-score 3.5 (SD 0.4) in those younger than 18 years	Single-arm trial Partially blinded Setmelanotide for 52 weeks, with dose up-titration (0.5–3.0 mg for those younger than 18 years, 1.0–3.0 mg for adults)	Baseline weight: 131.7 kg (SD 32.6) One-year weight: 115.0 kg (SD 29.6) Relative weight loss: −12.5% (SD 8.9, 90%CI –16.1 to −8.8, *p* < 0.0001), regardless of age BMI decrease in those younger than 18 years: Z-score −0.5 (SD 0.4, 90%CI –1.1 to 0.1, *p* = 0.14, *n* = 3) BMI decrease in adults: −5.2 kg/m^2^ (SD 3.9, 90%CI –8.1 to −2.3, *p* = 0.01, *n* = 7) *Hunger score* (11-point Likert scale): 7.0 (SD 0.8) at baseline → 4.1 (SD 2.1) at follow-up in those aged 12 years or older *Adverse effects*: skin hyperpigmentation, injection site reaction, nausea, vomiting
(71) ClinicalTrials.gov NCT02896192	POMC (*n* = 9) and PCSK1 (*n* = 1) deficiency, Hom or compound Het 73% female Age at inclusion ≥6 years Mean age 18.4 years (SD 6.2) Of whom, 6 were younger than 18, and 2 were younger than 12 y Obesity (>95th percentile, or adult BMI ≥ 30 kg/m^2^) Mean 40.4 kg/m^2^ (9.0) in adults Mean BMI Z-score 3.4 (0.6) in those younger than 18 years	Single-arm trial Partially blinded Setmelanotide for 52 weeks, with dose up-titration (0.5–3.0 mg for those younger than 18 years, 1.0–3.0 mg for adults)	Baseline weight: 115.0 kg (SD 37.8) One-year weight: 83.1 kg (SD 21.4) Relative weight loss: −25.6% (SD 9.9, 90% CI –28.8 to −22.0, p < 0.0001), regardless of age BMI decrease in those younger than 18 years: Z-score −1.6 (SD 0.9, 90%CI –2.3 to −0.9, *p* = 0.006, *n* = 6) BMI decrease in adults: −9.3 kg/m^2^ (SD 6.9, 90% CI –17.4 to −1.2, *p* = 0.07, *n* = 4) *Hunger score* (11-point Likert scale): 8.1 (SD 0.8) at baseline → 5.8 (SD 2.0) at follow-up in those aged 12 years or older *Adverse effects*: skin hyperpigmentation, injection site reaction, nausea, vomiting
(76) ClinicalTrials.gov NCT03651765	POMC deficiency, compound Het (*n* = 1) and Hom (*n* = 1) 2 adult women Age 21 and 26 at the start of treatment	Open-label extension study of setmelanotide 2.0 mg for 6.8–7.2 years Follow-up of (77) (ClinicalTrials.gov NCT02507492)	*Weight loss*: 35.8% (−55.6 kg) in patient 1; 47.5% (−72.6 kg) in patient 2 *BMI decrease*: BMI Z-score from 4.5 to 2.7 in patient 1; from 4.8 to 2.1 in patient 2 *Hunger scores* (11-point Likert scale): Pre-treatment 9–10 points Post-treatment 2–5 points See also (78) (QoL outcomes in LEPR-POMC deficiency)
(45) ClinicalTrials.gov NCT03651765	POMC (*n* = 3) or LEPR (*n* = 2) deficiency 4 male, 1 female Age at inclusion ≥15 years Mean age 23.8 years (range 15–33)	Open-label extension study of setmelanotide for 3–4 years	*Hunger scores* (11-point Likert scale) Pre-treatment 7–9 points (possible recall bias, some at the maximum 10 points) Post-treatment 2–7 points
Bardet-Biedl Syndrome			
(72) ClinicalTrials.gov NCT03746522	BBS / Alström syndrome → results in this table only relate to BBS (*n* = 32) 53% female Age at inclusion ≥6 years 15 adults +16 younger than 18 + 1 withdrawal Median age 17.5 years (IQR 12.0–25.5) Obesity (>97th percentile, or adult BMI ≥ 30 kg/m^2^)	Double blind 14-week RCT of setmelanotide up-titration to max 3.0 mg vs. placebo + Open-label follow-up for 52 weeks	*All those younger than 18 years* BMI: 37.4 kg/m^2^ (SD 9.4) → 34.2 kg/m^2^ (SD 10.1) BMI Z-score: 3.7 (SD 1.3) → 3.0 (SD 1.5), i.e., −0.8 (SD 0.5) over 52 weeks *Adults* BMI: 46.4 kg/m^2^ (SD 5.9) → 43.3 kg/m^2^ (SD 7.2) BMI decrease: −4.2 kg/m^2^ (SD 3.3), i.e., -9.1% (SD 6.8) over 52 weeks *Hunger score*: −30.5% (26.5%) *Adverse effects*: skin hyperpigmentation, injection site erythema, nausea, vomiting See also (73) (QOL improvements in BBS)
(46) ClinicalTrials.gov NCT03013543 and NCT03746522	BBS (*n* = 8 patients) 75% female Age at inclusion ≥15 years Mean age 36 years (range 17–65)	Setmelanotide for an average 29 months (range 12–48)	*Hyperphagia*: substantial improvement within 2 months of starting setmelanotide *Hunger scores* (11-point Likert scale) Pre-treatment 8–10 points (recall bias?) Post-treatment reduction −2 to −6 points
Ongoing pediatric studies			
ClinicalTrials.gov NCT04966741	Pediatric population from 2 to <6 years old Bi-allelic variants of POMC, PCSK1 or LEPR deficiency; or BBS	Phase 3 Setmelanotide for 52 weeks, with dose up-titration (0.5–2.0 mg for children)	*Active study, recruitment completed* Primary outcomes: BMI decrease in % and proportion of participants with at least 0.2 BMI Z-score reduction

Numbers are reported as mean (SD) unless stated otherwise. BBS, Bardet-Biedl Syndrome; CI, confidence interval; Het, heterozygous; Hom, homozygous; LEPR, leptin receptor; PCSK1, proprotein convertase subtilisin/kexin type 1; POMC, pro-opiomelanocortin; QoL, Quality of life; RCT, randomized controlled trial.

### Side effects/contraindication of setmelanotide

As with any injectable medication, hypersensitivity to setmelanotide or its excipients can be observed. Additionally, due to cross-stimulation of MC1R in melanocytes, some patients experience skin hyperpigmentation and darkening of naevi (51, 72). For safety, regular skin monitoring and restricted prescription through specialized centers are recommended. The clinical and research community now awaits long-term data on this second-generation MC4R agonist and the potential development of new therapies.

### Broader indications for setmelanotide?

Setmelanotide has been approved by the FDA for patients over 6 years of age with POMC, PCSK1, LEPR deficiencies and BBS, while the EMA has approved it for biallelic POMC, PCSK1, LEPR deficiencies and BBS in Europe (51, 74). Recently, its approval expanded to include patients as young as 2 years old (Clinicaltrials.gov no. NCT04966741). Trials are also underway to assess its efficacy in acquired hypothalamic obesity (75) and in other leptin-melanocortin pathway genes, including *SH2B1*, *CPE*, and 16p11.2 chromosomal rearrangements (51).

### Upcoming developments and outlook

With the high prevalence of obesity and advancing insights into its mechanisms, we anticipate drug developments targeting additional genes, alongside investigations into how different mutation types (e.g., null, frameshift, missense) affect treatment outcomes. Distinctions in receptor function—complete versus partial loss—may also yield varied drug responses (66). Given the diversity of outcomes in prior studies (Table 1), a standardized hunger scale, trial design, and extended follow-up are essential. In the development of MC4R agonists, we expect next-generation drugs to avoid MC1R cross-activity, thereby minimizing skin and naevi darkening, which currently necessitates regular monitoring. With the strong development GLP-1/GIP/glucagon receptor agonists, we expect some efficacy for individuals with monogenic and syndromic obesity in terms of weight loss and metabolic improvement (51).

## Conclusion

Obesity is a heterogenous disorder, involving single genes to hundreds of genes (2, 4, 5). Improved and precision treatment is now required. Recent advances in understanding the mechanisms leading to obesity and controlling appetite, hunger, and satiety have led to the development of drugs that can override genetic defects, enabling precision treatment.

## Footnotes

^1^ https://www.orpha.net/en/disease/detail/64?name=Alstrom%20syndrome%20&mode=name

^2^ https://redc.integromics.fr/surveys/index.php?s=XEFPD474YT
